# Structural Motifs Critical for *In Vivo* Function and Stability of the RecQ-Mediated Genome Instability Protein Rmi1

**DOI:** 10.1371/journal.pone.0145466

**Published:** 2015-12-30

**Authors:** Jessica A. Kennedy, Salahuddin Syed, Kristina H. Schmidt

**Affiliations:** 1 Department of Cell Biology, Molecular Biology, and Microbiology, University of South Florida, Tampa, Florida, 33620, United States of America; 2 Graduate Program in Cell and Molecular Biology, H. Lee Moffitt Cancer Center, Tampa, Florida, 33612, United States of America; 3 Cancer Biology and Evolution Program, H. Lee Moffitt Cancer Center, Tampa, Florida, 33612, United States of America; Consejo Superior de Investigaciones Cientificas, SPAIN

## Abstract

Rmi1 is a member of the Sgs1/Top3/Rmi1 (STR) complex of *Saccharomyces cerevisiae* and has been implicated in binding and catalytic enhancement of Top3 in the dissolution of double Holliday junctions. Deletion of *RMI1* results in a severe growth defect resembling that of *top3Δ*. Despite the importance of Rmi1 for cell viability, little is known about its functional domains, particularly in Rmi1 of *S*. *cerevisiae*, which does not have a resolved crystal structure and the primary sequence is poorly conserved. Here, we rationally designed point mutations based on bioinformatics analysis of order/disorder and helical propensity to define three functionally important motifs in yeast Rmi1 outside of the proposed OB-fold core. Replacing residues F63, Y218 and E220 with proline, designed to break predicted N-terminal and C-terminal α-helices, or with lysine, designed to eliminate hydrophobic residues at positions 63 and 218, while maintaining α-helical structure, caused hypersensitivity to hydroxyurea. Further, Y218P and E220P mutations, but not F63P and F63K mutations, led to reduced Rmi1 levels compared to wild type Rmi1, suggesting a role of the C-terminal α-helix in Rmi1 stabilization, most likely by protecting the integrity of the OB-fold core. Our bioinformatics analysis also suggests the presence of a disordered linker between the N-terminal α-helix and the OB fold core; a P88A mutation, designed to increase helicity in this linker, also impaired Rmi1 function in vivo. In conclusion, we propose a model that maps all functionally important structural features for yeast Rmi1 based on biological findings in yeast and structure-prediction-based alignment with the recently established crystal structure of the N-terminus of human Rmi1.

## Introduction

The RecQ-like DNA helicase family is evolutionarily conserved and necessary for genomic stability from bacteria to humans. In yeast the RecQ-like Sgs1 helicase forms a complex with the topoisomerase Top3 and the recQ-mediated genome instability 1 protein Rmi1 (STR) and facilitates both early and late steps of DNA double-strand break (DSB) repair [1–3]. Early in the repair of a two-ended DSB, STR contributes to DSB end resection to facilitate the formation of a single-strand 3’ overhang on which the homologous recombination (HR) factor Rad51 filament assembles [4–7]. This Rad51 filament is then able to initiate a genome-wide search for sequence homology, eventually leading to the formation of double Holliday Junctions (dHJs) that need to be resolved prior to cell division. Resolution can be achieved by the HJ-specific endonuclease Yen1, randomly leading to crossover and noncrossover products, or dHJs can be dissolved by STR in a process involving dHJ migration and decatenation of the single strands, yielding exclusively noncrossover products [8]. STR has also been implicated in the unwinding of strand invasion after extension of the invading 3’end by DNA synthesis to promote DSB repair by synthesis-dependent strand annealing, as well as reversal of strand invasion prior to 3’end extension [9]. Through these functions, STR promotes noncrossover outcomes of HR and regulates HR levels. Hence, yeast cells that lack the helicase activity of the STR complex (*sgs1Δ*) are prone to hyper-recombination, accumulate gross chromosomal rearrangements, and are hypersensitive to DNA-damaging agents, with cells lacking Top3 or Rmi1 additionally exhibiting a severe growth defect not seen in cells lacking Sgs1 [1,3,10,11]. Similarly, in mice inactivation of RMI1 or Topo IIIα leads to embryonic lethality earlier (no blastocysts) than inactivation of BLM (day 13.5), the human RecQ-like helicase most closely related Sgs1 [12].

Despite the growth defect of the *rmi1Δ* mutant, the contribution of Rmi1 to the function of the STR complex and the regions of Rmi1 that are critical for these functional contributions are still poorly understood. Rmi1, was first discovered in *S*. *cerevisiae* in a screen for components of the Sgs1/Top3 pathway [3]. Yeast cells lacking Rmi1 are hypersensitive to hydroxyurea (HU) and several other genotoxic agents, have an increased rate of spontaneous DNA damage as indicated by an increase in Rad52 foci, an increase in chromosomal rearrangements, and deficiency in activation of the DNA-damage checkpoint kinase Rad53 [1,3]. Diploids lacking Rmi1 are defective in meiosis, and deletion of genes with roles in the checkpoint response to replication stress, such as Mrc1, Tof1, Csm3, cause synthetic lethality, implying a diverse role for Rmi1 in several chromatin processes [1,3]. Despite the severity of *rmi1Δ* phenotypes, Rmi1 has no known catalytic function. It has been shown to stimulate the final decatenation step of dHJ dissolution by Sgs1/Top3, while inhibiting the relaxation activity of the topoisomerase on negatively supercoiled DNA, possibly by affecting the conformation of the topoisomerase gate [13–15]. This function is conserved in the BLM/Topo IIIα/Rmi1/Rmi2 (BTR) complex, the human variant of STR, and studies in human cell lines also imply a role for Rmi1 in Topo IIIα stability [16–18].

The N-terminal 219 residues of the 625-residue long human Rmi1 have been crystallized, providing some clues to its role in catalytic enhancement and stability of the BTR complex [19,20]. The N-terminus of human Rmi1 is most closely related to *S*. *cerevisiae* Rmi1, is capable of binding BLM and Topo IIIα, and contains an oligonucleotide-binding (OB) fold that is similar in structure to that of the replication protein A subunit RPA70, though it is suggested that it is incapable of binding DNA like RPA [20,21]. Human Rmi1 contains a disordered loop needed for dHJ dissolution enhancement of Topo IIIα [19]. Co-crystallization of the Rmi1 N-terminal lobe peptide with Topo IIIα reveals that the OB-fold of Rmi1 lies opposite of the ssDNA-binding domain of Topo IIIα, and a mostly disordered loop that protrudes from the OB fold of Rmi1 physically interacts with the topoisomerase by inserting itself into the central topoisomerase gate [22]. It has been hypothesized that this loop may be what facilitates the catalytic enhancement of Topo IIIα by regulating opening and closing of the gate [19,20].

Because of the severe growth retardation, low viability and rapid accumulation of suppressor mutations, the identification and functional analysis of deleterious *rmi1* mutations through genetic screens has proved difficult; one *rmi1* mutant, the temperature-sensitive E69K, was identified through this conventional approach [23]. In an effort to better understand the molecular basis of Rmi1 function, we have combined structure prediction tools with an *in vivo* mutational analysis of *RMI1* function in yeast. This approach has identified structural motifs that are essential for Rmi1 function and stability and we propose hypotheses for how these motifs contribute to Rmi1’s role in maintaining the functional integrity of the STR complex. As an alternative to primary sequence alignments, which have been only minimally informative because of the poor sequence conservation among Rmi1 homologues, we present a structure-based alignment between yeast and human Rmi1 that maps the location of conserved motifs and suggests differences in the size and structure of the DUF1767 domain.

## Materials and Methods

### Bioinformatics analysis

The 241 residues of *S*. *cerevisiae* Rmi1 and the N-terminal 240 residues of the 625-residue human Rmi1 were analyzed with algorithms for helical propensity [24,25], structural disorder using a combination of three predictors in VL-XT [26,27], and amino acid sequence alignments based on phylogenetic analysis in PhylomeDB v4 [28].

### Plasmids

The open reading frame of *RMI1* plus 500 bp up- and downstream was amplified by PCR from the endogenous *RMI1* locus of KHSY1338 (*ura3-52*, *leu2Δ1*, *trp1Δ63*, *his3Δ200*, *lys2-Bgl*, *hom3-10*, *ade2Δ1*, *ade8*, *sgs1*::*HIS3*, *YEL069C*::*URA3*,). The fragment was inserted into *Xba*I-digested pRS415 by gap-repair cloning using the non-homologous-endjoining deficient yeast strain KHSY2331 (*ura3-52*, *leu2Δ1*, *trp1Δ63*, *his3Δ200*, *lys2ΔBgl*, *hom3-10*, *ade2Δ1*, *ade8*, *YEL069C*::*URA3*, *lig4*::*loxP-G418-loxP*) and standard lithium-acetate transformation [29]. The integrity of *RMI1* and the promoter region in the resulting plasmid pKHS621 was verified by sequencing. Point mutations were introduced into the *RMI1* ORF in pKHS621 by QuikChange site-directed mutagenesis (Agilent Technologies). To construct pKHS621 derivatives that express myc-epitope-tagged Rmi1 and rmi1 mutant proteins, pKHS621 was linearized with *Box*I and the *HIS3*-linked myc-epitope-coding sequence from pFA6a-13MYC-HIS3MX6 [30] was inserted by gap-repair cloning. Point mutations were introduced into the resulting plasmid (pKHS630, S1 Table Plasmids used in this study) using the QuikChange protocol (Agilent Technologies).

### Hydroxyurea hypersensitivity assay

Derivatives of pKHS621 were transformed into KHSY4695 (*MAT*α, *ura3Δ0*, *leu2Δ0*, *his3Δ1*, *lys2Δ0*, *rmi1*::*HIS3*, *TOP3*.*V5*.*VSV*.*KANMX6*), grown to OD_600_ = 0.5 in synthetic complete media lacking leucine (SC-Leu), and spotted in 10-fold dilutions on yeast extract/peptone/dextrose (YPD) and on YPD supplemented with 150 mM hydroxyurea. Growth was documented after 3 to 5 days of incubation at 30°C.

### Viability assay

Yeast strain KHSY4695 (*rmi1Δ)* was transformed with plasmids pKHS621, pKHS624, and pKHS626. Independent cultures were set up from 9 to 12 transformants for each plasmid and grown to approximately 2 x 10^7^ cells/ml. Actual cell counts were determined using a hemocytometer and cultures were diluted to plate ~ 400 cells. Plates were incubated for 3 days at 30°C and colonies counted. Viability was calculated by dividing the number of colony forming units by the number of cells plated based on the hemocytometer count.

### Protein extraction and Western blotting

A BY4741 derivative (*MAT*a, *ura3Δ0*, *leu2Δ0*, *his3Δ1*, *lys2Δ0*, *RAD51-V5-6xHIS*.*KANMX6)* from the Yeast Cross and Capture Collection (GE Dharmacon) was transformed with pKHS630 or its derivatives (S1 Table Plasmids used in this study) expressing myc-tagged Rmi1 or rmi1 mutants and grown in synthetic complete media lacking histidine overnight. Cultures were then diluted to OD_600_ = 0.2 and grown to OD_600_ = 0.4. Cultures were synchronized in G1 phase with 2 μg/ml of α-factor for one hour, followed by addition of 1 μg/ml of α-factor for a second hour. Cells were released for 30 min and cells equivalent to 2 ODs were harvested and washed twice in distilled water. Whole cell extract was prepared with 20% trichloroacetic acid as previously described [31], separated by SDS-PAGE, and myc-tagged Rmi1 and rmi1 mutants detected by Western blotting on PVDF and hybridization with c-myc (9E10) monoclonal antibody (Covance).

## Results

### Computational analysis of yeast Rmi1 structure

**Fig 1:**
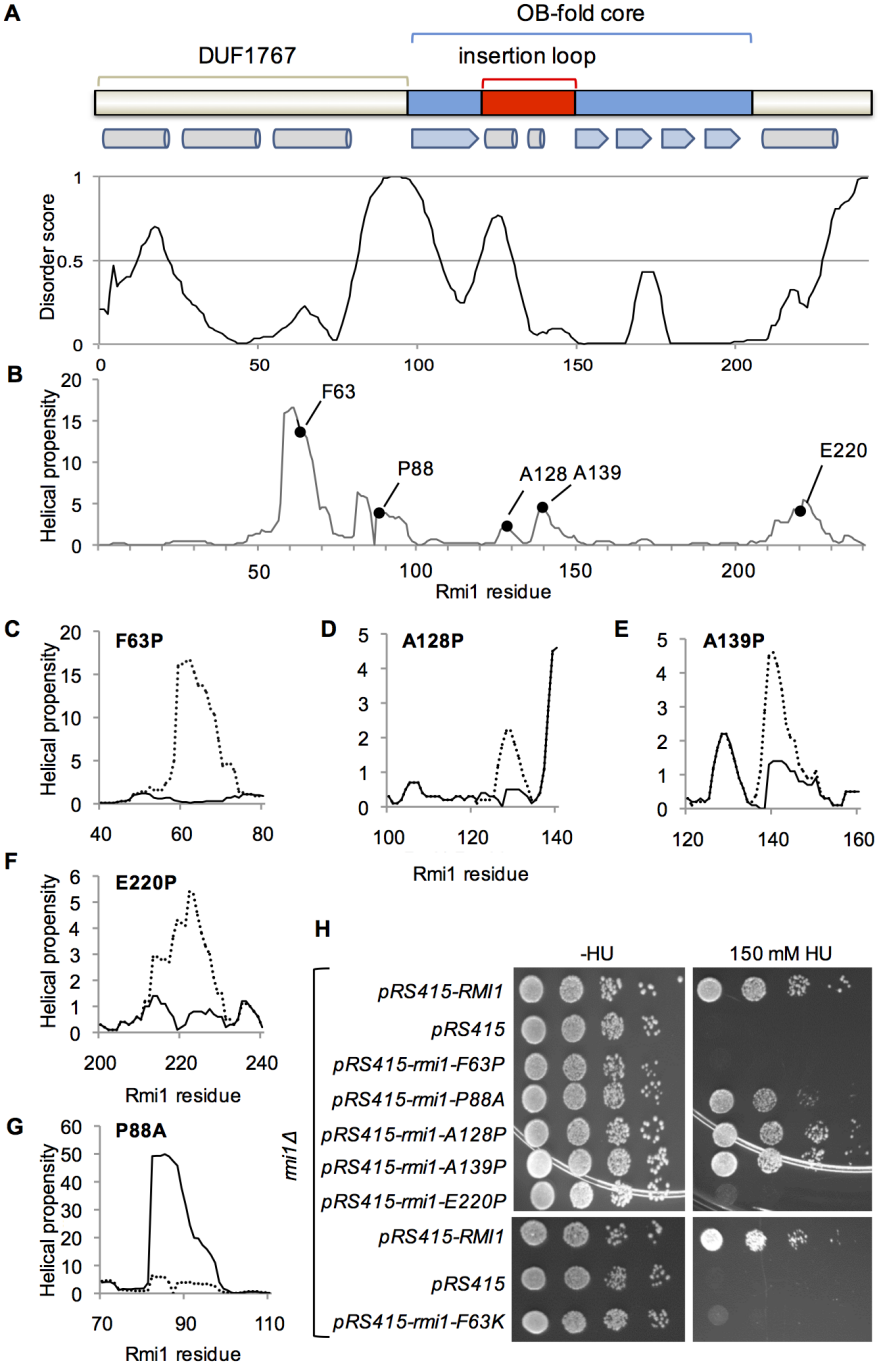
Structure-prediction-guided mutagenesis of *S*.*c*. Rmi1. A, prediction of order/disorder in Rmi1 by the VLXT algorithm [26,27]. A score of 1 denotes an ideal prediction of disorder and a score of 0 an ideal prediction of order with the order/disorder threshold at a score of 0.5. A domain of unknown function (DUF1767), and an OB-fold with an insertion loop are conserved in all Rmi1 species [39]. The position of DUF1767, the OB-fold, the insertion loop and a predicted flexible linker between DUF1767 and the OB-fold shown above the disorder plot are based on the VLXT order/disorder prediction. α-helices (cylinders) and β-strands (arrows) in this region of human Rmi1 are indicated below the domain map. B, prediction of four regions of increased helical in Rmi1 with residues F63, A128,A139, E220 having some of the highest helical propensity in the DUF1767 domain, the insertion loop, and the C-terminus, respectively. We predict that P88 is responsible for the sudden loss in helical propensity in the linker that connects N-terminal domain and the OB-fold. C–G, substitution of F63, A128, A139 and E220 with proline, which has the lowest helical propensity of all amino acids, is predicted to disrupt the increased helical propensity in these regions, whereas substitution of P88 with alanine, which has excellent helical propensity, is predicted to lead to a strong increase in continuous helical propensity of the linker. H, plasmid pRS415 expressing *RMI1* and *rmi1* mutants under control of the endogenous *RMI1* promoter were transformed into *Δrmi1* mutant KHSY4695 and tested for the ability to suppress the hypersensitivity of the *rmi1Δ* strain to hydroxyurea.

Determining functionally important residues in *S*. *cerevisiae* Rmi1 has been challenging as it lacks catalytic activity and a crystal structure has not been resolved. The primary sequence is only minimally conserved (~35% identical residues between yeast genera, 18% between *S*. *cerevisiae* and human Rmi1) and lengths range from 241 residues in *S*. *cerevisiae* to 625 residues in humans. The crystal structure of the N-terminal 219 residues of human Rmi1 was recently resolved [20]. It revealed an N-terminal three-helix bundle of unknown function (DUF1767) followed by an OB-fold with a largely unstructured loop inserted between strands β1 and β2 by which Rmi1 binds to Topo IIIα. In the absence of catalytic activity/domains and very limited sequence identity, we reasoned that structure prediction tools [25,32,33] could reveal functionally important motifs in yeast Rmi1. Analyzing the distribution of ordered and disordered residues, we noticed disordered N- and C-termini as well as two internal regions of increased disorder (Fig 1A). Further, we identified two regions of increased helical propensity, spanning residues 58–74 and residues 212–228 near the N- and C-terminal disordered regions, as well as two regions of lesser helical propensity between residues 125–132 and 137–145 (Fig 1B). Alignment of these two predictors would be consistent with the putative OB-fold core mapping to the central, ordered region, and the topoisomerase-binding loop to the disordered insertion with two segments of weak helical propensity. The DUF1767 domain, predicted to be present in Rmi1 of all species, is typically located N-terminally of the OB-fold core, and appears to be connected to it in yeast Rmi1 by a disordered linker (Fig 1A).

### Mutational analysis of predicted structural elements of yeast Rmi1

We had previously determined that disruption of an α-helix was most effective when a residue with high helical propensity near the peak or in the N-terminal half of the helix was replaced with the helix breaker proline [31]. Therefore, to determine the importance of the four regions of increased helicity for Rmi1 function, we constructed F63P, A128P, A139P and E220P mutations. The proline substitutions led to marked decreases in predicted helical propensity in these regions (Fig 1C–1F). We also noticed that the proline at position 88 seemed to disrupt what might otherwise be a region with high helical propensity, and hypothesized that this native break was helping to maintain a degree of flexibility in what would otherwise be a persistent, structured region. We considered that replacing P88 with a residue with high helical propensity that was otherwise benign, such as alanine, would restore helicity to this region. Indeed, the P88A mutation is predicted to lead to an extraordinary increase in helical propensity not seen in any region of the wildtype forms of yeast or human Rmi1 (Fig 1G). We exploited the HU hypersensitivity of yeast cells lacking Rmi1 [1] to assess the functional impact of these proline substitutions *in vivo*. We found that *rmi1Δ* cells expressing rmi1-A128P and rmi1-A139P exhibited the same HU sensitivity as the *rmi1Δ* mutant complemented with wildtype *RMI1*, whereas the *rmi1-P88A* allele was only able to partially suppress the HU hypersensitivity of *rmi1Δ* (Fig 1H). This mild, 5- to 10-fold, increase in HU sensitivity of the rmi1-P88A mutant compared to wildtype or the rmi1-A128P mutant was not due to decreased viability of the rmi1-P88A mutant as the viability of all three strains was similar (33–36%).

The *rmi1-F63P* allele caused the same degree of HU hypersensitivity as a deletion of *RMI1*, indicating that it was a null allele (Fig 1H). We also considered the possibility that the phenotype of the F63P mutation could be due to the loss of a strong hydrophobic interaction via the aromatic residue. Thus, we chose to replace F63 with a hydrophilic residue with high helical propensity, such as lysine, that would be predicted to maintain the structural integrity of the motif, but change its chemical properties. We found that the F63K mutation caused the same hypersensitivity to HU as the F63P mutation (Fig 1H), implicating that this residue maps to an α-helical structure that must conserve both its shape and hydrophobic character in order to maintain wildtype function of Rmi1. Similar to F63, proline substitution of E220 in the predicted C-terminal helix abolished Rmi1 function *in vivo* (Fig 1F and 1H).

**Fig 2:**
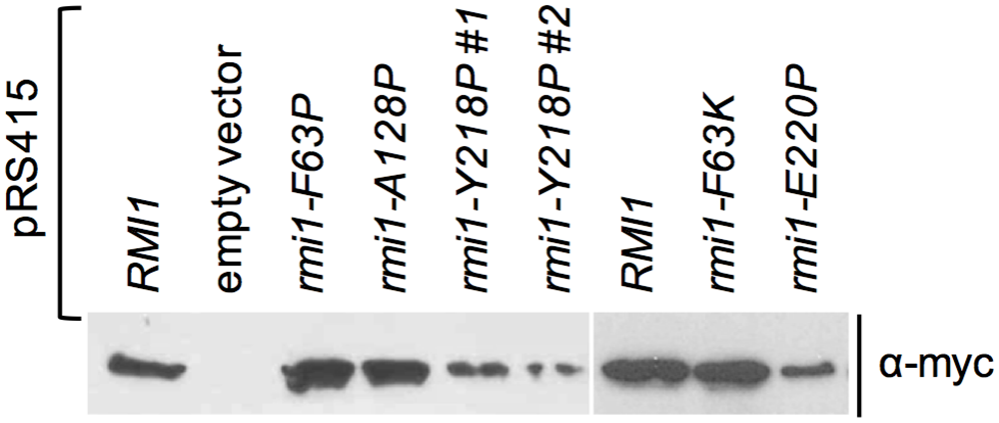
Effect on expression levels of mutations designed to disrupt structural motifs of yeast Rmi1. Mutants of Rmi1 with point mutations that disrupt the function of the putative N-terminal α-helix (F63P, F63K) are expressed at similar levels as wildtype Rmi1 and the benign rmi1-A128P mutant, whereas mutations that disrupt the function of the putative C-terminal α-helix (Y218P, E220P) are expressed at reduced levels. Whole cells extracts from equal numbers of cells from synchronized cultures expressing myc-epitope-tagged Rmi1 or rmi1 mutants were analyzed by Western blotting. Expression levels of two independently constructed plasmids expressing rmi1-Y218P are shown.

The function of the putative helices defined by F63 and E220 may arise from stabilizing the putative OB-fold core of Rmi1 as seen in other proteins containing this fold type [34] or by otherwise contributing to Rmi1 stability. To test this possibility, we inserted a myc-epitope-coding sequence at the 3’ end of *RMI1* in pRS415 and introduced the deleterious F63P, F63K and E220P mutations, as well as the benign A128P mutation as a control. Equal numbers of cells were harvested from synchronized cultures for protein extraction and Western blotting, which revealed that mutations in the C-terminal helix, but neither in the N-terminal helix nor the helical elements in the insertion loop led to reduced Rmi1 levels (Fig 2).

### Structure predictions and in vivo mutagenesis suggest differences in the N-termini of yeast and human Rmi1

**Fig 3:**
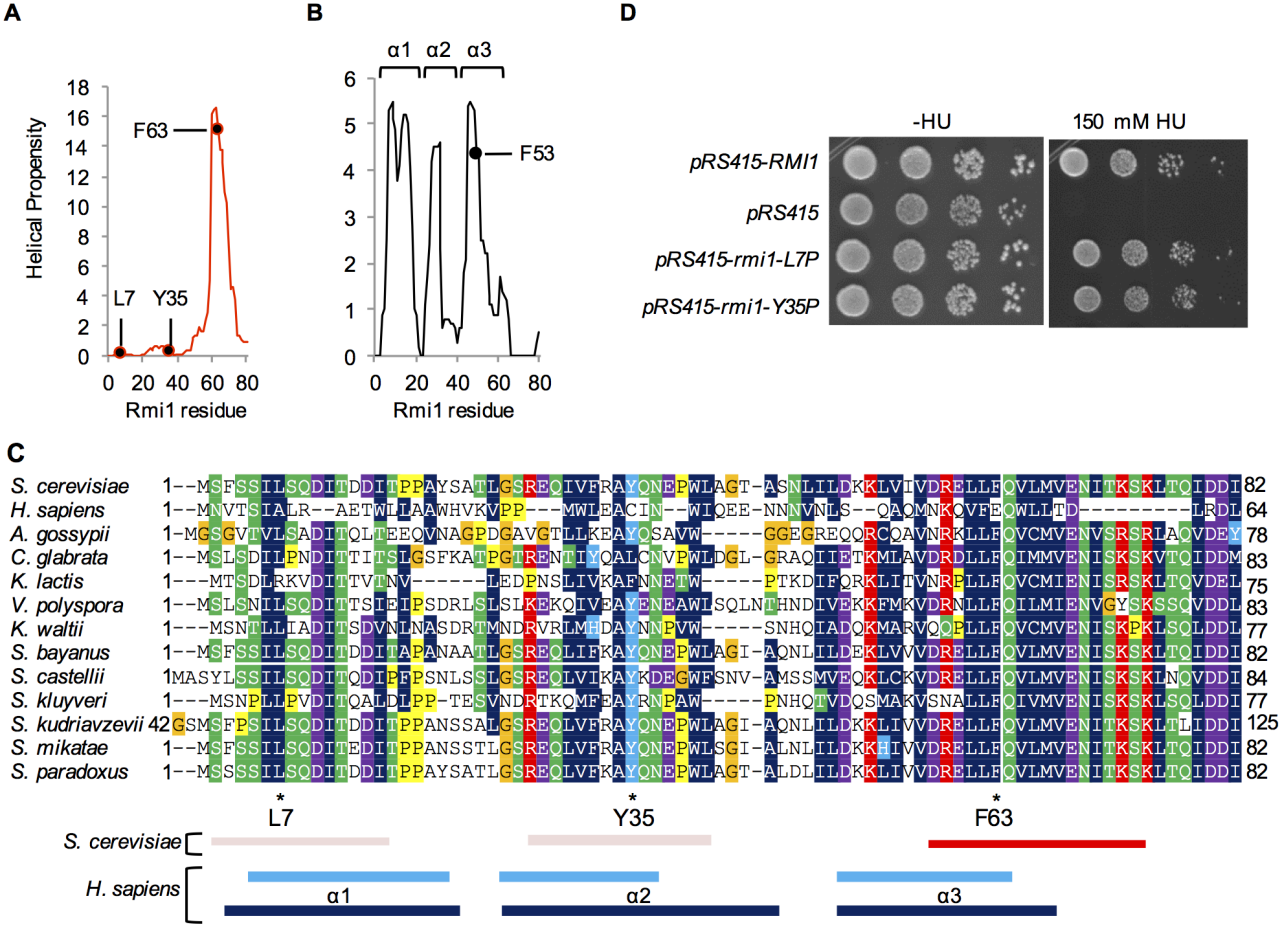
Structural prediction and *in vivo* functional analysis suggest differences between the N-termini of yeast and human Rmi1. A and B, one segment of increased helical propensity (residues 58–74) is predicted for the N-terminus of yeast Rmi1, whereas three such segments (residues 5–18, 23–32, 44–55) are predicted for human Rmi1, corresponding to the three-helix bundle confirmed in the Rmi1 crystal structure [4CGY [19]]. The estimated equivalent to yeast F63, F53 in α3 of human Rmi1, is indicated. C, alignment of Rmi1 N-termini from twelve yeast species in PhylomeDB [28] suggests two segments of conserved residue chemistry between residues 2–15 and residues 27–41 (indicated below the alignment by light red rectangles), in addition to the highly conserved segment predicted to have high helical propensity (indicated by a red rectangle). The N-terminus of human Rmi1 was aligned to *S*.*c*. Rmi1 by ClustalW and manually adjusted. The three segments of increased helical propensity predicted in the DUF1767 region of human Rmi1 are shown as light blue rectangles (residues 5–18, 23–32, 44–55) and dark blue rectangles (residues 3–19, 23–38, 44–58) indicate the confirmed location of α-helices that make up the three-helix bundle in the DUF1767 domain in the crystal structure of the N-terminus of Rmi1 with Topo IIIα (4CGY) [19]. D, substitution of L7 and Y35 with proline, aimed at preventing the two segments from adopting α-helical structure induced by intra- or intermolecular binding events, does not impair Rmi1 function *in vivo*.

Structural prediction analysis indicated a single α-helical region in the N-terminus of yeast Rmi1, centered on F63 (Fig 3A). When we extended this analysis to the N-terminus of human Rmi1, we identified three segments of increased helical propensity (Fig 3B), which form a three-helix bundle in the crystal structure [19,20]. The lack of helical propensity in the first 57 residues of yeast Rmi1 suggests that this region may not adopt helical structures in the apo form as human Rmi1 does. To identify conserved residues and regions of conserved chemical character that could be indicative of a functional role, we analyzed primary sequence alignments of Rmi1. We found that *S*.*c*. Rmi1 is ~85% identical to Rmi1 of other *Saccharomyces* species, but identity markedly decreased to ~30% when compared to yeast species outside of the genus (e.g., *K*. *lactis*, *C*. *glabrata*), and to ~18% when compared to the N-terminal 241 residues of human Rmi1. Because of the low level of sequence identity between human and yeast Rmi1 we decided to analyze the alignment of the N-termini of twelve closely related Rmi1 sequences from fully sequenced *Saccharomyces* and non-*Saccharomyces* yeast species in PhylomeDB v4 [28]. We noticed two discrete regions (Fig 3C, residues S2-I15 and R27-L41 of *S*.*c*. Rmi1) that contain hydrophobic residues in the *i*,*i*+4 pattern typical of an α-helix and are separated from each other by residues with the lowest helical propensity, proline and glycine. To test the possibility that these two regions could become helical upon binding to another protein, possibly Sgs1, or could be analogous to α1 and α2 in human Rmi1, we replaced L7 and Y35 with proline (Fig 3C). Expression of either mutant, however, was sufficient to fully restore wildtype growth to the *rmi1Δ* mutant on HU (Fig 3D), suggesting either that, unlike in human Rmi1, this region in yeast Rmi1 does not adopt α-helical structures or that any helical structure or binding-induced folding in this region is not required for Rmi1’s role in tolerating HU-induced DNA-damage. Based on comparisons of helical propensity and primary sequences of yeast Rmi1 and the N-terminus of human Rmi1 (Fig 3A–3C), we propose structural equivalence between the sole predicted α-helix in yeast and α3 in human Rmi1, with a potential equivalent of F63 at residue F53 in human Rmi1.

### A conserved alpha helix in the C-terminus contributes to yeast Rmi1 stability

**Fig 4:**
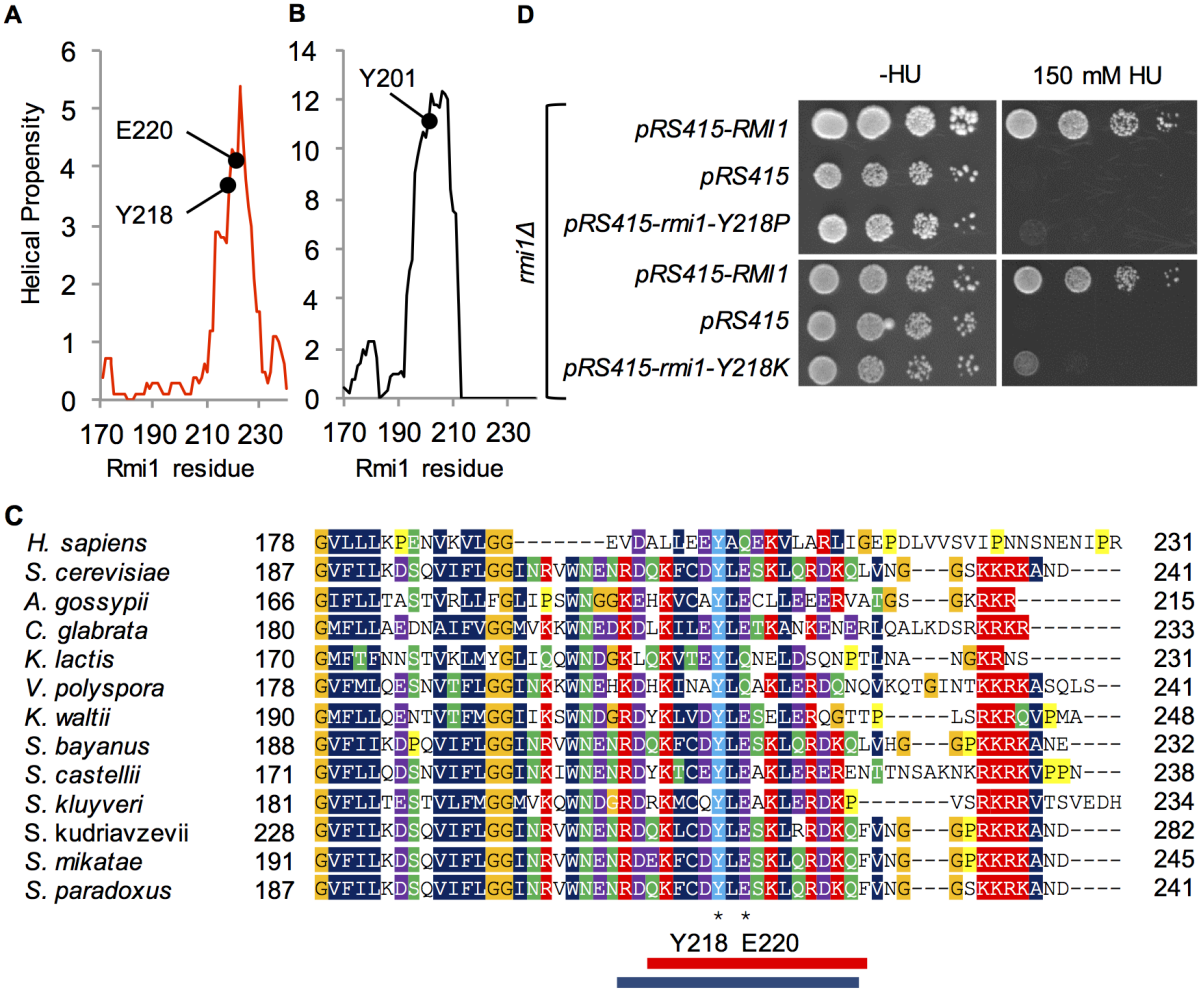
Mutational analysis of the C-terminal region of increased helical propensity. A and B, Yeast Rmi1 and human Rmi1-N, the N-terminal 240-residue region of human Rmi1 that is most similar to yeast Rmi1, have comparable predicted structure in the far C-terminus. Y218 is in the same predicted helix as E220 and has a potential equivalent in Y201 in human Rmi1. C, PhylomeDB alignment of Rmi1 C-termini from different yeast species reveals a highly conserved tyrosine at position 218. The corresponding region of human Rmi1 (residues 178–231) was manually aligned to the yeast Rmi1 phylome. The position of the predicted α-helix in yeast Rmi1 is indicated by a red rectangle below the alignment. The corresponding α-helix defined by Y201 in the crystal structure of human Rmi1 (PDB: 4CGY) is indicated by a blue rectangle. D, like *rmi1-E220P*, *rmi1-Y218P* and *rmi1-Y218K* mutants fail to complement the hydroxyurea hypersensitivity of an *Δrmi1* mutant.

When we extended the analysis of sequence alignments to the C-terminus of Rmi1 it revealed that the chemical characteristics of the predicted α-helical region centered on residue E220 were conserved, with a short stretch of hydrophobic residues surrounded by charged residues (Fig 4C). Whereas neither E220 nor the acidic or hydrophilic character of the residue was conserved outside of the *Saccharomyces* genus, the hydrophobic residues were, including a tyrosine at position 218. We hypothesized that this residue was not only part of the functional α-helical structure we had inferred from the E220P mutant, but was also a key residue for binding in an otherwise fairly charged α-helix. Indeed, we found that either breaking the helix (rmi1-Y218P) or increasing its hydrophilicity (rmi1-Y218K) abolished Rmi1 function (Fig 4D). Although yeast and human Rmi1 are only 18% identical, we found that they share regions of similar helical propensity, including the region that surrounds Y218 in yeast and Y201 in an amphipathic α-helix in human Rmi1 (Fig 4A–4C). Similar to the disruption of the C-terminal helix by the E220P mutation, the Y218P mutation led to reduced Rmi1 levels (Fig 2).

Taken together, the bioinformatics analysis and corresponding mutagenesis of Rmi1 *in vivo* indicates the presence of two functionally critical N-terminal and C-terminal α-helices, with the latter contributing to Rmi1 stability. It further indicates a disordered linker, defined by the P88A mutation, that appears to connect the N-terminal α-helix to the putative OB-fold core. The function of a disordered loop in the OB fold whose equivalent in human Rmi1 binds Topo IIIα, was not disrupted by mutagenesis of two predicted, albeit very weak, helical motifs.

## Discussion

In this study, we have combined three bioinformatics tools—order/disorder prediction, helical propensity and phylomes [24,25,27,28,32,33]–with *in vivo* mutagenesis to elucidate structure/function relationships in yeast Rmi1. We focused on the regions that surround a putative OB-fold core previously identified in human Rmi1 that is involved in binding BLM and Topo IIIα [22,35]. The regions surrounding the central OB-fold core are predicted to contain two short regions of increased helical propensity (residues 58–74, 212–228) and a highly disordered linker (residues 82–97) connecting the N-terminal helix to the OB-fold core. We determined that the structural and chemical integrity of the N-terminal α-helix defined by F63P/K, and the C-terminal α-helix defined by E220P and Y218P/K, are critical for Rmi1 function *in vivo*, and that the disorder of the linker, defined by P88A, contributes to normal Rmi1 function.

**Fig 5:**
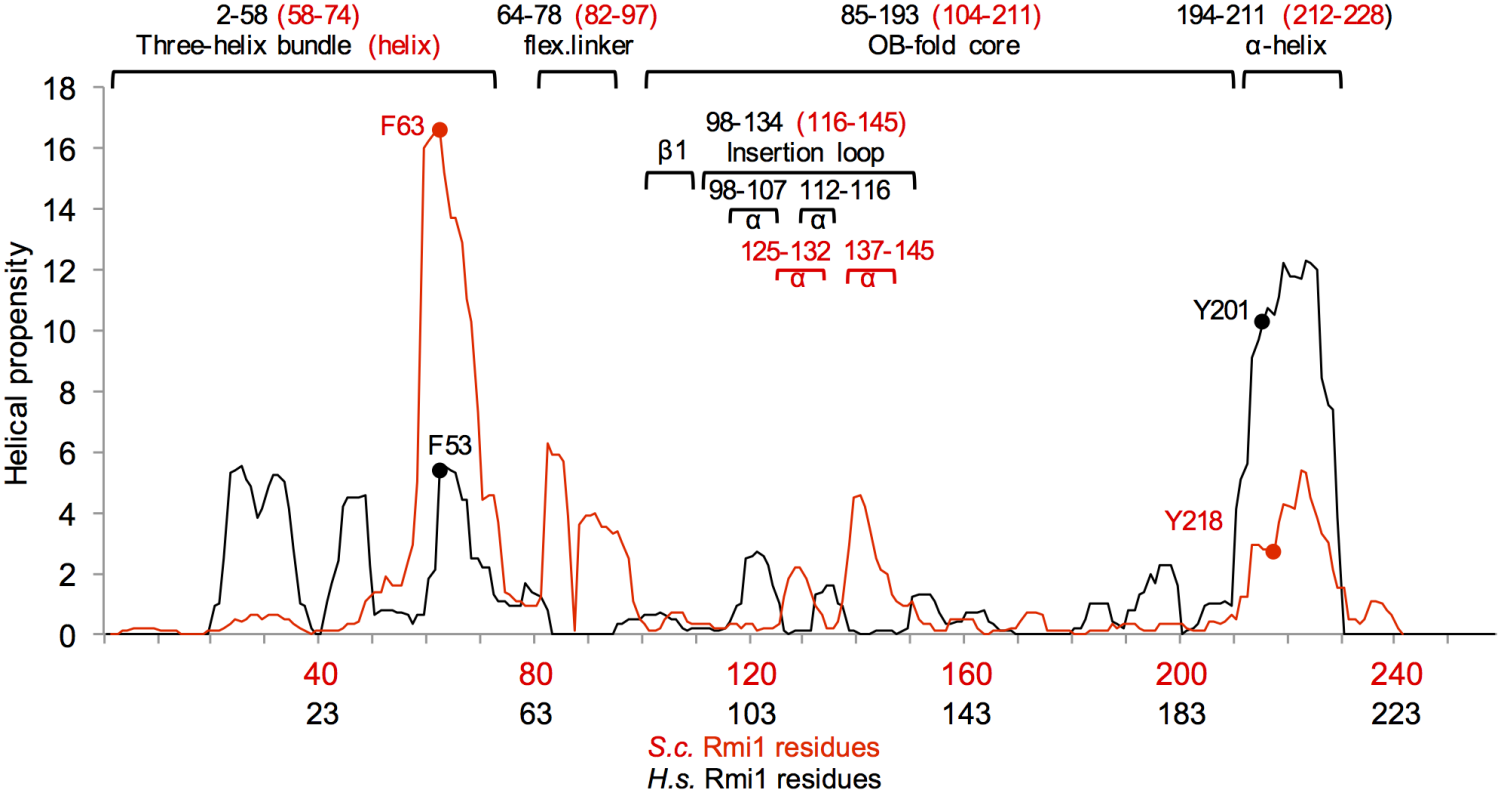
Proposed structure-prediction-based alignment of yeast and human Rmi1. Residues to which confirmed domains in the human Rmi1 crystal structure (4CGY) map are indicated above the alignment in black. Proposed location of conserved domains and motifs in yeast Rmi1 are indicated in red. Conserved residues whose mutation to proline abolished Rmi1 function *in vivo* are indicated in the alignment with the proposed corresponding residue in human Rmi1 (F63/F53; Y281/Y201). Compared to human Rmi1, the N-terminus (DUF1767) of yeast Rmi1 appears to be extended by approximately 18 residues. The predicted size and location of the topoisomerase-binding loop (residues 116–145) differs from that previously proposed (residues 87–145) for yeast Rmi1 [19].

**Fig 6:**
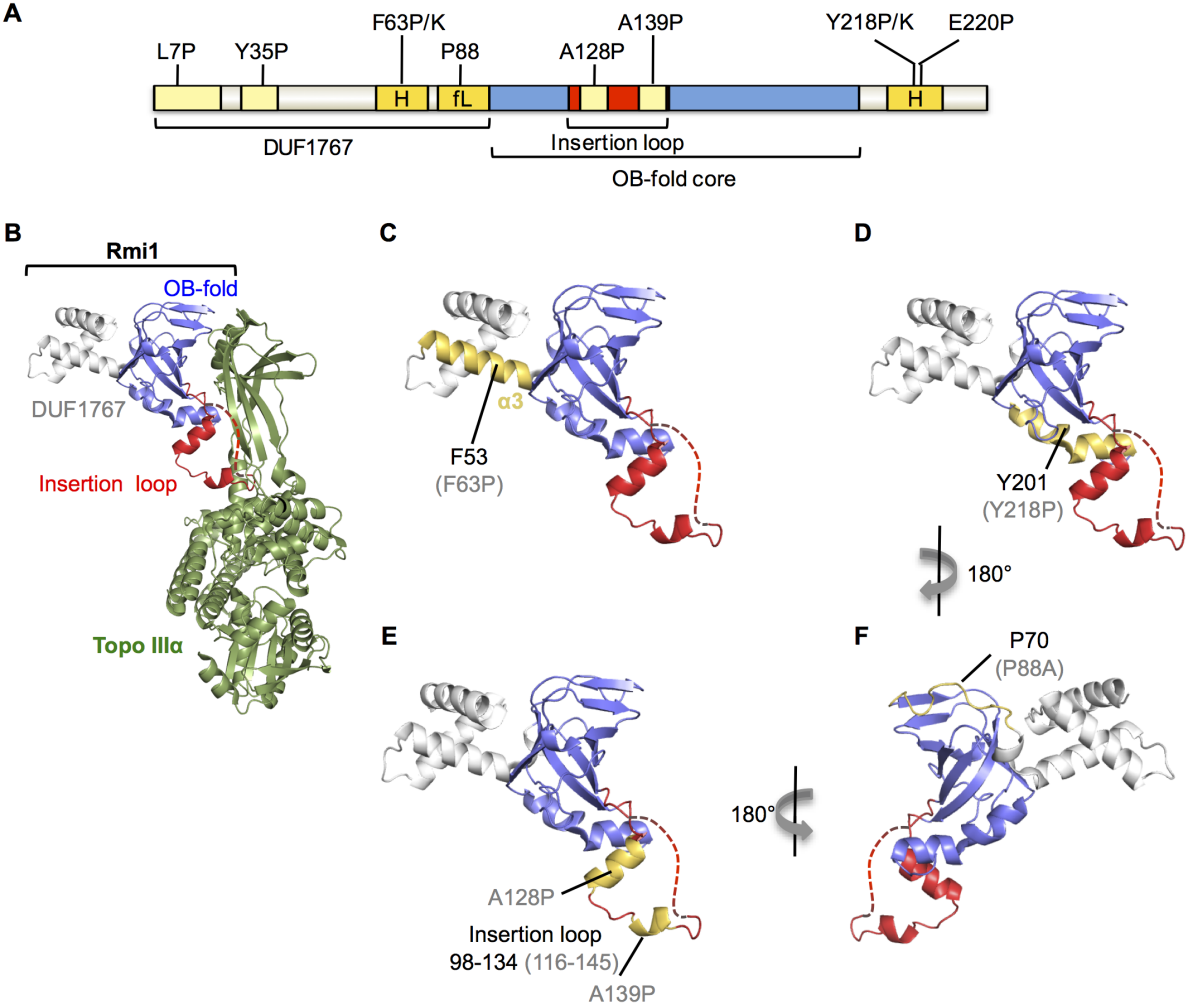
Conserved domains, and putative differences between yeast Rmi1 and the N-terminus of human Rmi1. A, functional importance of seven structural motifs predicted in yeast Rmi1 was tested by analyzing point mutations *in vivo*. Mutation of putative motifs highlighted in light yellow did not impair Rmi1 function *in vivo*, including L7P and Y35P mutations in the putative DUF1767 domain, and A128P and A139P in the topoisomerase-binding loop. Mutations in motifs highlighted in dark yellow impaired Rmi1 function *in vivo*; F63P and F63K mutations in an N-terminal α-helix (H), Y218P, Y218K, and E220P mutations in a C-terminal α-helix (H) caused null phenotypes, and the P88A mutation in the flexible linker (fL) between the DUF1767 domain and the OB-fold caused intermediate functional impairment. B, he crystal structure of the N-terminus of human Rmi1 bound to Topo IIIα (*4CGY* [19]) was rendered in PyMol. The DUF1767 domain, the OB-fold core and the insertion loop extending between strands β1 and β2 are shown in grey, blue, and red, respectively. A part of the disordered insertion loop missing from the crystal structure is indicated by a red dashed line. Topo IIIα is shown in green. C, the domain structure of Rmi1 is shown as in (B) with α3, corresponding to the functionally important, putative α-helix in yeast Rmi1, highlighted in yellow. Residue F53 of human Rmi1 and the corresponding F63P null mutation in yeast Rmi1 (in brackets) are indicated. D, the C-terminal α-helix interacting with the bottom of the OB-fold core is shown in yellow. Y201 and the corresponding Y218P null mutation in yeast Rmi1 (in parentheses) are indicated. Disruption of this α-helix leads to lower Rmi1 expression levels and total loss of Rmi1 function (Fig 3E). E, human Rmi1 contains two helical segments in the insertion loop, shown in yellow. A128P and A139P mutations were designed to disrupt helical propensity in the corresponding insertion loop in yeast Rmi1, but did not interrupt Rmi1 function. F, a flexible linker connects the DUF1767 domain to the OB-fold in human Rmi1, indicated in yellow. A single proline at position 88 is predicted to disrupt the strong helical propensity of this linker in yeast Rmi1 whereas this linker is more proline-rich in human Rmi1. The location of P70 in human Rmi1, corresponding to P88 in yeast Rmi1, is indicated.

Performing the same structural prediction analysis for human Rmi1 suggests that the two N- and C-terminal α-helices and the spacing between them, where the core of an OB-fold has been confirmed in human Rmi1, are conserved (Fig 5). In human Rmi1, a loop that maps to residues 98–134 emerges from the OB-fold between strands β1 and β2 and inserts itself into the Topo IIIα gate; its deletion eliminates complex formation of Rmi1 with BLM and Topo IIIα [20]. Based on a sequence alignment Bocquet and colleagues [19] suggested that the equivalent loop for Top3 binding in yeast Rmi1 maps to residues 87–146 and showed that replacement of this region with a scrambled version of equal chemistry still mediated binding to Sgs1 and Top3, but failed to stimulate Top3 catalytic activity and dHJ dissolution. The structural alignment in our study, however, which we explored because of the poor sequence conservation of only 18% between yeast and human Rmi1, suggests that residues 87–116 of yeast Rmi1 contain the β1 strand of the OB-fold and the disordered linker that connects β1 to the N-terminal helical region (Fig 5). The insertion loop in yeast Rmi1, therefore, may be significantly shorter, mapping to residues 116–145. As in human Rmi1, this insertion loop contains two segments of increased helical propensity (Figs 1B and 6E), but proline mutagenesis of these segments (A128P, A139P) suggests that the adoption of helical structure is not required for Rmi1’s function. Since both regions adopt short helices, if any, and the prolines replaced alanines that are likely to be in the first turn, it is also possible that proline substitution is not structurally disruptive in this disordered loop.

The N-terminal region flanking the OB-fold in the crystal structure of human Rmi1 forms a three-helix bundle that has been designated a DUF1767 domain [22]. The three α-helices are also indicated in our structural analysis, whereas the corresponding region in yeast Rmi1 contains only one predicted α-helix (residues 58–74). Our structural alignment (Fig 5) suggests that it corresponds to α3 of human Rmi1 (Fig 6C); this is also supported by our manual alignment of the N-terminus of human Rmi1 with the N-terminal sequences of the yeast Rmi1 phylome (Fig 3C). How this α-helix contributes to yeast Rmi1 function is unclear. Genetic analysis of Rmi1 from *Arabidopsis thaliana* showed that the OB-fold core and the N-terminal helical region DUF1767 can function independently of each other [36]. In human Rmi1, the three-helix bundle in the corresponding DUF1767 region makes contacts with the top region of the OB-fold core and was shown to be indispensable for its folding and solubility [22]. The F63P mutation, which we designed to disrupt the N-terminal α-helix in yeast Rmi1, caused a null phenotype; however, it had no noticeable effect on Rmi1 levels in the cell. This is in contrast to the C-terminal α-helix whose amphipathic properties and importance for Rmi1 stability strongly suggest that it protects the OB-fold core. Instead, the N-terminal α-helix—and the region N-terminal of the OB-fold in general—could also mediate association with another protein, possibly Sgs1, whose binding site on Top3/Rmi1 is unknown. This type of small binding motif paired with disorder has been seen in other proteins, including yeast Adr1, which contains two small zinc finger motifs in a disordered domain [37]; interestingly, the disordered components of this domain undergo extensive folding when contact is made between the zinc fingers and DNA. The structural alignment between yeast and human Rmi1 put forth in this study also suggests that the N-terminus of yeast Rmi1 is extended by approximately 18 residues. Since residues 1–16 are arranged in the typical *i*,*i+4* pattern of an α-helix (residues F3, L7, I11, I15) and the yeast phylome indicates two regions of similarity in the N-terminal 57 residues, we tested if binding-induced helix formation could also be a function of the extended unstructured N-terminus of yeast Rmi1, but found that introducing proline residues where prospective helices might form (L7, Y35), did not impair Rmi1 function. This limited analysis, however, cannot exclude the possibility that shorter helices fully capable of supporting Rmi1 function *in vivo* can still be induced with the L7P and Y35P mutations present.

In contrast to the N-terminal α-helix, our findings suggest that the C-terminal α-helix defined by the Y218P/K and E220P mutations plays a major role in stabilizing Rmi1 as seen in other proteins containing an OB-fold [34]. Our structural prediction suggests that the C-terminal α-helix extends from residues 212 to 228 and is equivalent to the α-helix between residues 194–211 in human Rmi1, with Y201 corresponding to Y218 in yeast Rmi1 (Figs 4A–4C and 5). In contrast to mutations that disrupt the N-terminal helix, the Y218P and E220P mutations cause substantially reduced Rmi1 levels, which is the most likely cause of their null phenotype. Indeed, in the crystal structure of human Rmi1 the hydrophobic face of the corresponding amphipathic α-helix packs against the bottom of the OB-fold core, which is likely to stabilize it by shielding it from the solvent (Fig 6D) [19]. Binding of human Rmi1 to Rmi2 involves extensive interactions between the α-helices C-terminal of the second OB-fold of Rmi1 and the OB-fold of Rmi2 [22,38]; however, a similar function in mediating interaction with another protein has not been identified for the α-helix C-terminal of the N-terminal OB-folds of human or yeast Rmi1.

Finally, our bioinformatics analysis suggests a disordered loop in yeast Rmi1 linking the OB-fold core to an N-terminal α-helix. The structural disorder in this linker appears to depend largely on a single proline, whereas multiple prolines are present in human Rmi1, suggesting a more flexible linkage of the three-helix bundle to the OB-fold core (Fig 6F). A mutation predicted to increase helicity of this flexible linker partially impaired Rmi1 function. This mutant grows normally, but exhibits increased DNA-damage sensitivity, most likely by reducing the overall conformational elasticity of Rmi1.

## Supporting Information

S1 Table Plasmids used in this study. (PDF)
