# Supplementary material for: Structural Motifs Critical for In Vivo Function and Stability of the RecQ-Mediated Genome Instability Protein Rmi1
Source: PLoS One. 2015 Dec 30;10(12):e0145466. doi: 10.1371/journal.pone.0145466 (PMC4696737; doi:10.1371/journal.pone.0145466)
Supplement: S1 Table — (PDF) [file pone.0145466.s001.pdf]

S1 Table. Plasmids used in this study

Plasmid	Description
pKHS621	*pRS415-RMI1*
pKHS622	*pRS415-rmi1-F63P*
pKHS635	*pRS415-rmi1-F63K*
pKHS623	*pRS415-rmi1-E220P*
pKHS624	*pRS415-rmi1-A128P*
pKHS625	*pRS415-rmi1-A139P*
pKHS626	*pRS415-rmi1-P88A*
pKHS627	*pRS415-rmi1-Y218P*
pKHS634	*pRS415-rmi1-Y218K*
pKHS628	*pRS415-rmi1-L7P*
pKHS629	*pRS415-rmi1-Y35P*
pKHS630	*pRS415-RMI1-myc.HIS3MX6*
pKHS631	*pRS415-rmi1-F63P-myc.HIS3MX6*
pKHS632	*pRS415-rmi1-A128P-myc.HIS3MX6*
pKHS633	*pRS415-rmi1-Y218P-myc.HIS3MX6*
pKHS642	*pRS415-rmi1-F63K-myc.HIS3MX6*
pKHS643	*pRS415-rmi1-E220P-myc.HIS3MX6*
